# Neonatal nurses’ perceptions of providing palliative care in neonatal intensive care units in Saudi Arabia

**DOI:** 10.1097/MD.0000000000049821

**Published:** 2026-07-17

**Authors:** Hassan Al-Shehri, Ashwaq Alotaibi, Bayan Alanazi, Abdulelah Alshehri, Albandari Alajlan, Musab Alsulami, Razan Alshehri, Fahad Alshehri, Reemas A. Alshammari, Khawlah Alrashed, Reem Alhumaidan, Atheer Al-Ghadeer, Ahmed M. Almutairi, Abdullah Alzayed

**Affiliations:** a Department of Pediatrics, College of Medicine, Imam Mohammad Ibn Saud Islamic University (IMSIU), Riyadh, Saudi Arabia; b College of Medicine, Imam Mohammad Ibn Saud Islamic University (IMSIU), Riyadh, Saudi Arabia.

**Keywords:** neonate, NICU, nurses, palliative care, perception

## Abstract

There is limited literature on the perceptions and practices of nurses in Saudi Arabia related to neonatal palliative care (NPC). This study aimed to assess NPC perceptions and practices among female nurses in Saudi Arabia. This cross-sectional survey study was conducted in Saudi Arabia between May and October 2025. The inclusion criteria for this study were nurses working in neonatal intensive care units in Saudi Arabia, regardless of the level of care provided. Factors associated with higher perception scores were identified using multivariable logistic regression analysis. A total of 170 nurses were included in this study. The majority worked in the governmental sector (148, 87.6%). Most participants reported that their institutions had formal NPC guidelines (142, 83.5%). Perception scores showed a significant difference by nurses’ education level (*P* = .01). Nurses with a bachelor’s degree or lower reported a mean score of 85.16 ± 16.64, whereas those with a master’s degree or PhD had a mean score of 71.44 ± 31.30 (*P*-value = .02). Participants aged between 30 and 40 years showed lower odds of attitude (adjusted odds ratio = 0.33, 95% confidence interval: 0.11–1.00, *P* = .05), which was borderline significant. This study assessed NPC among nurses in neonatal intensive care units in different settings in Saudi Arabia. The results suggested the importance of education in both NPC awareness and practice. Further studies are needed to confirm these findings. Improving nurses’ knowledge and skills may lead to better palliative care for neonates and their families.

## 1. Introduction

Neonatal palliative care (NPC) focuses on meeting the physical, emotional, social, and spiritual needs of newborns who require specialized care.^[1]^ The main goals of NPC are relieving pain, managing distressing symptoms, and improving the overall quality of life for both infants and their families. NPC extends beyond treating and managing adverse symptoms to include emotional guidance and support for families after the death of an infant.^[2]^

Infants in neonatal intensive care units (NICUs) can benefit from combined neonatal and palliative team efforts, especially in critical cases.^[3]^ Additionally, NPC includes supporting pregnant females who are facing serious conditions by combining medical knowledge with emotional support from the first day of diagnosis to the end of their child’s life.^[4]^ Furthermore, the application of clinical practice guidelines in multiple care environments helps to prepare for the outcome and improves decision-making.^[5–7]^

In Saudi Arabia, nurses in neonatal settings showed a low level of knowledge regarding NPC practice. A study conducted among 200 nurses in a tertiary center showed that most of the nurses did not receive any education related to palliative care and had only a moderate level of experience in all areas of NPC.^[8]^ These results could be explained by low training levels, emotional barriers associated with caring for dying children, and the absence of specific guidelines in these types of settings.^[9]^ In recent years, the importance of palliative care in NICUs has increased significantly, but there remains significant room for improvement in Saudi Arabia regarding nurses’ NPC perceptions and practices. Globally, there are multiple studies that have assessed nurses’ knowledge, attitude, barriers, and practice; however, in Saudi Arabia, there is limited research, and NPC perceptions and practices remain underexplored. This study aimed to assess the NPC perceptions and practices among female nurses in Saudi Arabia. This may help identify educational gaps that can be addressed to improve palliative care practices and neonatal outcomes.

## 2. Methods

### 2.1. Study design

This was a multicenter cross-sectional survey study conducted in Saudi Arabia between May and October 2025.

### 2.2. Study population and settings

The inclusion criteria for this study were nurses working in NICUs in Saudi Arabia, regardless of the level of care they provided. Nurses with no experience in the NICU were excluded. The study participants were recruited using a convenience sampling technique. This sampling technique involved selecting accessible participants who were willing to participate and met the study inclusion criteria.

### 2.3. Study outcomes

The questionnaire comprised 2 sections. The first section examined demographic and professional characteristics such as age, hospital of practice, duration of experience in the NICU, education, current workplace, and whether the institution had formal NPC guidelines. The second section examined the attitudes of NICU nurses toward NPC by utilizing an adapted version of the Neonatal Palliative Care Attitude Scale, with permission obtained for use and modification of the scale.^[10]^ This scale utilized a 5-point Likert scale ranging from strongly disagree to strongly agree.

### 2.4. Questionnaire reliability and validity

The questionnaire tool was examined through a pilot study involving a group of NICU nurses who confirmed that the questions accurately reflected the participants’ clinical practice and were easily understandable. No modifications were made to the original scale. The psychometric properties of the adapted questionnaire were evaluated to determine its internal reliability and validity before the primary data analysis. The correlation matrix was suitable for factor analysis, as evidenced by the statistically significant results of Bartlett’s test of sphericity (χ^2^ = 3060.708, df = 253, *P* < .001) and the Kaiser–Meyer–Olkin measure of sampling adequacy (0.915), which exceeded the recommended threshold of 0.70. The 23-item scale was subjected to principal component analysis with Varimax rotation. Four components were extracted, collectively accounting for 70.70% of the total variance, the eigenvalues of which exceeded 1.0. Component 1, which consisted of 14 items that reflected unit-level palliative care support and practice, accounted for 42.46% of the variance. Personal attitudes and emotional burden were captured by component 2, which accounted for 17.82% of the variance. Components 3 and 4 accounted for 5.29% and 5.12% of the variance, respectively. These covered conflicting care obligations and exposure to mortality. The structural validity of the scale was supported by all items exhibiting factor loadings of at least 0.40 on their principal component. The extracted components demonstrated adequate item representation, as evidenced by the communalities, which ranged from 0.508 to 0.834. The internal reliability of the scale was examined using Cronbach’s alpha test, which measured 0.917 and demonstrated excellent internal consistency.

### 2.5. Ethical approval

This study was approved by the Institutional Review Board at Al-Imam Muhammad Ibn Saud Islamic University (IMSIU), Riyadh, Saudi Arabia (Project number 797/2025). All participants provided their informed consent before participating in the study. This study was conducted in accordance with the World Medical Association Declaration of Helsinki.

### 2.6. Patient and public involvement

It was not appropriate or possible to involve patients or the public in the design, conduct, reporting, or dissemination plans of our research.

### 2.7. Sample size

This was an exploratory study with no formal sample size calculation. All eligible participants who met the inclusion criteria were included in this study. The achieved sample size was considered appropriate for our exploratory analysis that examined 6 predictor variables using multivariable logistic regression analysis.

### 2.8. Data analysis

The analyses were performed using the Statistical Package for the Social Sciences version 28 (IBM Corporation). The demographic characteristics, such as the age group and hospital of practice, were reported as frequencies and percentages. The continuous data, such as the scale, were expressed as means and standard deviations. The median of the scale was reported as 88 (interquartile range: 80.00–94.00) and utilized as a cutoff point to perform multivariable logistic regression (adjusting for potential confounders: age, hospital of practice, years of experience in a NICU, education, current workplace, and whether the institution had a formal NPC guideline). The distribution of the perception score was negatively skewed (skewness = −1.482, SE = 0.186), which substantially deviated from normality (skewness ratio = −7.96, exceeding the ±1.96 threshold). Due to the skewness of the perception score, the median score was deemed a more appropriate measure of central tendency and was used for dichotomizing the perception score.^[11,12]^ The independent variables were the demographic characteristics, and the results were reported as adjusted odds ratios (aORs) and 95% confidence intervals, identifying variables associated with higher perception scores. An analysis of variance was undertaken, and if significant, a post hoc test was applied. All data were normally distributed as confirmed by the Kolmogorov–Smirnov test. All analyses were two-tailed, and a *P*-value < .05 was considered statistically significant. No missing data were identified in the dataset for this analysis.

## 3. Results

A total of 170 nurses were involved in this study. The demographic characteristics of the included nurses are presented in Table 1. Most nurses were aged 30 to 40 years (79, 46.5%), while 58 were aged 20 to 30 years (34.1%). Most nurses held a bachelor’s degree (135, 79.4%), while 26 (15.3%) had a diploma. The majority worked in the governmental sector (148, 87.6%). Al-Iman General Hospital had the largest representation (51, 30.2%), followed by the National Guard Hospital (33, 19.5%). In terms of NICU experience, 55 nurses (32.4%) had 1 to 5 years, and 47 nurses (27.6%) had 6 to 10 years. Most participants reported that their institutions had formal NPC guidelines (142, 83.5%).

**Table 1:** Demographic and practice characteristics of neonatal nurses.

Demographic and professional characteristics	N (%)
Age (yr)	
20–30	58 (34.1)
30–40	79 (46.5)
40–50	25 (14.7)
Above 50	8 (4.7)
Hospital of practice	
King Salman Hospital	17 (10.1)
National Guard Hospital	33 (19.5)
King Saud Medical City	11 (6.5)
Al Yamamah Hospital	28 (16.6)
Al-Iman General Hospital	51 (30.2)
Specialized Medical Center Hospital	20 (11.8)
Prince Sultan Military	9 (5.3)
How long have you worked in a neonatal intensive care unit (yr)?	
<1	14 (8.2)
1–5	55 (32.4)
6–10	47 (27.6)
11–15	30 (17.6)
>15	24 (14.1)
Education	
Diploma	26 (15.3)
Bachelor’s degree	135 (79.4)
Master’s degree	8 (4.7)
PhD	1 (0.6)
Which describes your current workplace?	
Governmental sector	148 (87.6)
Private sector	21 (12.4)
Does your institution have a formal neonatal palliative care guideline?	
No	10 (5.9)
Yes	142 (83.5)
Unsure	18 (10.6)

Table 2 presents a comparison of the mean perception scores of neonatal nurses regarding palliative care provision, broken down by their demographic and professional characteristics. A significant difference in perception scores was observed by educational level (*P* < .05). Nurses with a bachelor’s degree or lower reported a mean score of 85.16 ± 16.64, while those with a master’s degree or PhD had 71.44 ± 31.30 (*P*-value = .02).

**Table 2:** The mean neonatal nurses’ perception scores stratified by demographic and practice characteristics.

Variables	Mean ± SD	*P*-value
Age (yr)		
20–30	88.79 ± 14.29	.08
30–40	80.94 ± 21.23	
40–50	85.16 ± 12.05	
Above 50	85.00 ± 13.99	
Hospital of practice		
King Salman Hospital	83.88 ± 22.98	.56
National Guard Hospital	85.48 ± 15.49	
King Saud Medical City	75.45 ± 26.12	
Al Yamamah Hospital	83.00 ± 20.59	
Al-Iman General Hospital	84.92 ± 13.45	
Specialized Medical Center Hospital	86.30 ± 18.39	
Prince Sultan Military	91.89 ± 15.93	
How long have you worked in a neonatal intensive care unit (yr)?		
<1	82.50 ± 22.08	.34
1–5	87.85 ± 17.90	
6–10	80.53 ± 19.57	
11–15	85.23 ± 17.56	
>15	84.33 ± 9.55	
Education		
Bachelor’s degree or lower	85.16 ± 16.64	.02
Master’s degree or PhD	71.44 ± 31.30	
Which describes your current workplace?		
Governmental sector	85.25 ± 17.01	.36
Private sector	81.57 ± 18.92	
Does your institution have a formal neonatal palliative care guideline?		
No	77.30 ± 11.44	.11
Yes	85.68 ± 18.00	
Unsure	78.56 ± 18.00	

SD = standard deviation.

Table 3 presents the results of the logistic regression analysis examining factors associated with neonatal nurses’ perceptions of providing NPC. None of the examined variables, including age, hospital of practice, years of NICU experience, and other demographic variables, showed statistically significant associations (*P* > .05). AORs ranged widely across categories, reflecting variability in perceptions among different subgroups, with participants aged 30 to 40 years achieving lower average perception scores (aOR = 0.33, 95% confidence interval: 0.11–1.00, *P* = .05), but at only a borderline significance level.

**Table 3:** Factors associated with higher perception scores.

Variables	Adjusted OR of having higher perception score (95% CI)*	*P*-value
Age (yr)		
20–30	Reference	
30–40	0.33 (0.11–1.00)	.051
40–50	0.66 (0.14–3.12)	.604
Above 50	0.70 (0.07–6.94)	.757
Hospital of practice		
King Salman Hospital	Reference	
National Guard Hospital	0.55 (0.14–2.20)	.399
King Saud Medical City	0.20 (0.03–1.59)	.129
Al Yamamah Hospital	1.56 (0.38–6.36)	.538
Al-Iman General Hospital	0.87 (0.23–3.28)	.832
Specialized Medical Center Hospital	2.17 (0.18–26.44)	.543
Prince Sultan Military	1.77 (0.26–12.32)	.561
How long have you worked in a neonatal intensive care unit (yr)?		
<1	Reference	
1–5	1.39 (0.37–5.26)	.625
6–10	0.78 (0.17–3.67)	.751
11–15	1.60 (0.26–9.95)	.615
>15	0.32 (0.04–2.53)	.281
Education		
Bachelor’s degree or lower	Reference	
Master’s degree or PhD	1.60 (0.25–10.36)	.619
Which describes your current workplace?		
Governmental sector	Reference	
Private sector	0.13 (0.01–1.47)	.100
Does your institution have a formal neonatal palliative care guideline?		
No	Reference	
Yes	3.18 (0.32–31.99)	.327
Unsure	1.15 (0.09–14.49)	.913

CI = confidence interval, OR = odds ratio.

* The multivariable logistic regression analysis adjusted for age, hospital of practice, years of experience in a neonatal intensive care unit, education, current workplace, and whether the institution had a formal neonatal palliative care guideline as potential confounders.

## 4. Discussion

The findings showed that most female nurses achieved a good NPC perception score. The mean NPC perception score varied by demographic characteristics, with diploma holders achieving higher scores compared with those with bachelor’s degrees. Although the aOR results showed no statistically significant associations for most variables, young nurses had a higher NPC perception score than older nurses.

The findings of our research are consistent with several recent studies that examined nurses’ views and attitudes toward NPC. Previous research has shown that education and institutional policies play an important role in shaping nurses’ comfort and preparedness to provide palliative care, although the relationship was not always statistically significant. For instance, Chin et al and Abuhammad et al reported that nurses who received formal training in palliative care or worked in a unit that applied a guideline showed increased confidence and positive attitudes toward providing palliative care.^[2,13]^ These results support the theory that education and policies help nurses to understand the principles of palliative care.

The similarity between our findings and previous studies may be attributed to shared challenges and developmental stages in NPC programs across healthcare systems. For instance, studies conducted in the Middle East and internationally showed that nurses have positive attitudes toward palliative care but varied preparedness levels. Previous literature showed that formal education and practical exposure improve nurses’ confidence and empathy when caring for terminally ill neonates, and that the absence of training is the main barrier to delivering palliative care.^[14,15]^ These similarities highlight the importance of training and workshops related to palliative care practices.

In contrast, some studies have reported stronger, statistically significant relationships between demographic characteristics and perceptions of NPC, which were not evident in the present study. This discrepancy could be explained by methodological and contextual factors such as the homogeneity of the current sample, which consisted entirely of female nurses from Riyadh. Limited variability in professional and institutional characteristics may have reduced the ability to detect significant associations. Furthermore, differences in measurement tools, sample size, and hospital resources can contribute to variation between studies. Another possible reason is that the availability of NPC guidelines does not necessarily mean effective application, and nurses may not always be aware of or trained to apply these policies in daily practice.

Overall, the comparison with prior studies suggests that education and institutional policies remain the most influential factors shaping nurses’ perceptions of NPC. The current results, though not statistically significant, do show some alignment with this evidence, suggesting that educational programs and workshops can improve nurses’ readiness to deliver high-quality end-of-life care for neonates and their families. Alqahtani et al found that supporting training programs and ensuring the practical application of guidelines could help fill the gap between knowledge and practices in Saudi NICUs and other healthcare settings.^[16]^

The results of this study support the importance of promoting neonatal nurses’ preparedness and confidence in providing palliative care within the NICU setting.^[17]^ Although no statistically significant differences were found between most demographic and professional variables, the observed trends suggest that training and continuous education programs could improve nurses’ perceptions and practices.^[18]^ Incorporating NPC principles into undergraduate and postgraduate nursing curricula in faculties, in addition to providing workshops and learning programs, may enhance nurses’ competence, comfort, and communication skills when caring for terminally ill neonates and their families.^[19–21]^ Based on these results, it is recommended that healthcare policymakers and nursing leaders strengthen ongoing education and training in NPC.

Furthermore, implementing clear guidelines for NPC practices across NICUs in Saudi Arabia would improve the quality of care for families and their children. Additionally, collaboration among healthcare providers in NICUs, such as physicians and psychological specialists, is essential to deliver effective palliative care. However, future studies should expand to assess nurses from regions beyond Riyadh and to involve other healthcare providers to identify gaps in practices and to support the development of NPC training courses and workshops.

The findings of this research highlighted that NPC guidelines are widely in place in Saudi Arabia. At the same time, the observed variability in NPC perception scores reflects the need for standardized nursing training concerning NPC. This highlights the importance of continuous medical education to enhance nurses’ NPC practices. This will improve the consistency and standardization of practice. Future longitudinal studies on a larger scale are needed to examine changes in nurses’ perceptions over time and examine causality across the study variables.

There were several potential limitations to this study. First, the sample included only female nurses from 1 city; the sample should be expanded to several settings in multiple cities to enhance the generalizability of the results beyond the studied population and enhance the external validity of the findings. Second, the study design itself posed limitations: cross-sectional surveys are prone to response bias, as the results are self-reported. Furthermore, self-administered research related to palliative care could be prone to social desirability bias, where participants’ responses reflect perceived professional norms rather than their true perceptions.

The use of convenience sampling might limit the generalizability of the study findings and introduce selection bias, as participants were selected based on accessibility and willingness to participate rather than through a systematic sampling strategy. Additionally, the sample diversity was limited; most of the nurses were younger. Older nurses should be included to assess the effect of nursing experience. Including physicians and other professionals will lead to a better understanding of the gaps among healthcare providers. The findings of the regression analysis did not identify a statistically significant association between participants’ baseline and practice characteristics and their perceptions of providing palliative care in the NICU. This should be confirmed in future research. The dichotomization of the perception score based on the median value for logistic regression analysis may have led to a loss of information compared with continuous variable analysis. To preserve statistical information, future research should recruit larger samples and analyze perception scores as continuous variables using nonparametric or robust regression approaches. The results of this study should be interpreted as hypothesis-generating rather than confirmatory, due to the cross-sectional design and the absence of statistically significant associations in the multivariable logistic regression analysis. This interpretation will serve as a foundation for future longitudinal research in NPC education.

## 5. Conclusion

This study assessed NPC perceptions among nurses working in NICUs in different hospitals in Saudi Arabia. Although the findings showed no significant association between the nurses’ demographics and their perceptions, the results suggested the importance of education in both NPC awareness and practice. To confirm this finding, further studies are needed. Additionally, a supportive work environment is essential to encourage nurses to participate in training, and clear guidelines ought to be provided that help families and improve their quality of life. Improving nurses’ knowledge and skills may lead to better palliative care for neonates and their families.

## Acknowledgments

We would like to thank Dr Victoria Kain for giving permission to use and modify the NiPCAS for this project.

## Author contributions

**Conceptualization:** Hassan Al-Shehri.

**Data curation:** Hassan Al-Shehri.

**Formal analysis:** Hassan Al-Shehri.

**Funding acquisition:** Hassan Al-Shehri.

**Investigation:** Hassan Al-Shehri, Ashwaq Alotaibi, Bayan Alanazi, Abdulelah Alshehri, Albandari Alajlan, Musab Alsulami, Razan Alshehri, Fahad Alshehri, Reemas A. Alshammari, Khawlah Alrashed, Reem Alhumaidan, Atheer Al-Ghadeer, Ahmed M. Almutairi, Abdullah Alzayed.

**Methodology:** Hassan Al-Shehri.

**Project administration:** Hassan Al-Shehri.

**Resources:** Hassan Al-Shehri, Ashwaq Alotaibi, Bayan Alanazi, Abdulelah Alshehri, Albandari Alajlan, Musab Alsulami, Razan Alshehri, Fahad Alshehri, Reemas A. Alshammari, Khawlah Alrashed, Reem Alhumaidan, Atheer Al-Ghadeer, Ahmed M. Almutairi, Abdullah Alzayed.

**Software:** Hassan Al-Shehri.

**Supervision:** Hassan Al-Shehri.

**Validation:** Hassan Al-Shehri.

**Visualization:** Hassan Al-Shehri.

**Writing – original draft:** Hassan Al-Shehri.

**Writing – review & editing:** Hassan Al-Shehri, Ashwaq Alotaibi, Bayan Alanazi, Abdulelah Alshehri, Albandari Alajlan, Musab Alsulami, Razan Alshehri, Fahad Alshehri, Reemas A. Alshammari, Khawlah Alrashed, Reem Alhumaidan, Atheer Al-Ghadeer, Ahmed M. Almutairi, Abdullah Alzayed.
